# Miscellany

**Published:** 1903-10

**Authors:** 


### Miscellany.

Formaldehyde for Pulp-Canal Cleansing.—MM. André and Marion recommend the following formula:

> Formic aldehyde, 40 parts;
> Essence of geranium, 20 parts;
> Alcohol, eighty per cent., 40 parts.

They claim that the alcohol, by uniting with and dissolving the fatty matter always present in a foul pulp-canal, assists very materially the coagulation of the albuminous contents by the formaldehyde. They also claim that until the pulp-canal is thoroughly clean formaldehyde is not a sterilizer; therefore its application should be continued after the cleanliness of the canal has been proved by the usual tests. The above formula has resulted from a long series of carefully conducted experiments, undertaken with the purpose of utilizing the valuable properties of this agent in treating putrescent conditions of the pulp-canal and its surroundings safely, efficiently, and with the least discomfort to the patient. —*British Journal of Dental Science,* June 1, 1900, page 481.

---

Chloroform Liniment for Dental Use.—

> Chloroform, ℥ii;

> Ether, ʒii;

> Alcohol, ℥i;

> Gum-camphor, ʒi.

This is a valuable preparation, and should be kept handy in every dental office. A lock of cotton, liberal in size, saturated with it and placed on the gum over a painful tooth, usually gives prompt relief. It is especially useful in painful conditions following an arsenical application, pulp-canal filling, the insertion of a crown or bridge, and the after-pain of tooth-extraction. It usually mitigates very much the pain attending an alveolar abscess, and is a comfort to the patient if used before beginning the preparation of a root for crowning. Its efficiency is increased by adding a small portion of chloretone immediately before making the application. With the addition of a very little formaldehyde and chloretone or cocain, it may be used to anæsthetize a pulp preparatory to its extirpation. When applied to the gums the first effect is usually a sharp and rather severe smarting, which passes off in a minute or two.—W. H. T.

---

Care of the Hands.—It is not often that attention is called in societies to the necessity of more care by the dentist, in regard to the hands and instruments. A case of infection that I recently had to trace up has proved to my mind that we all ought to be more careful than we are. The case was that of a young, healthy woman about twenty-five years of age, who was in the country, not exposed to infection of any kind. She went to a dentist and had some teeth filled. This dentist was a consumptive in pretty bad shape, and a few days after he finished his work her mouth became very sore and her teeth loose, and three weeks afterwards she had a hard cough, and rapidly developed all symptoms of consumption. Examination of the sputum and the lungs showed tuberculosis bacilla in the saliva, and a consolidation in the lung. It is generally difficult to trace the case to the man who is responsible for it; but to my mind there are many cases where infection is given by the dentist through his hands or instruments. I think the dentist's hands are often responsible. They do not clean them properly. I dare say among the members of this society you will not find a bichloride solution in one office out of fifty; and yet dentists handle consumptive cases, and go from one to the other, just washing their hands with soap and water. It is not fair to the patient, and I think it ought to be brought more fully before the dentists. If you use alcohol and glycerin between the washings, you can have your hands soft and nice at the end of the day.—Dr. Russell, Brooklyn, *Items of Interest.*

---

Beeswax for filling Roots.—Dr. B. F. Arrington has used best quality of beeswax for filling roots, with very satisfactory results, for many years. He coaxed it into roots with hot instruments.—*Dental Digest.*

SHARPENING LANCETS, EXCAVATORS, ETC.—Disks made from emery paper Nos. 00 and $\frac{1}{2}$, an inch and a half in diameter, are placed in alternate layers, to about a quarter of an inch in thickness, upon a disk made of tin slightly smaller in diameter than the emery disks and mounted on a screw mandrel.

The tin disk serves to hold the paper disks flat while sharpening the instrument. Place the mandrel in a hand-piece and hold it with the left hand, leaving the right free to apply the instrument to be sharpened in contact with the coarser grit, to trim instrument to desired shape.

Tear off this disk and finish the sharpening upon the finer grit. A keen, even edge can be given to the instrument in a very short time in this way.—DR. F. J. PATTERSON, *Dental Review.*

---

THE SANGER HALF-COLLAR CROWN.—Dr. R. M. Sanger, of East Orange, N. J., describes in the August, 1903, number of the *Dental Cosmos* a half-collar crown he has invented. He begins its construction by cutting a piece of pure gold plate No. 30 into a shape closely resembling a pointed finger-nail, or a rounded angle triangle with a base about half an inch long, the two sides a little longer. He grasps this by the base with a pair of clasp-benders, including a little more than the width of the intended collar, then bends the pointed portion over the convex beak of the clasp-benders and hammers it down with a small bench hammer. This shapes the gold, rudely, to the form of the intended cap, the excess of gold forming two tabs, one on each side; these are now cut off, leaving the front portion which is to form the top of the cap free to be burnished into close contact with the root. The cap is now adjusted to the root, reduced to a proper size, and bent and burnished to an accurate fit. This constitutes all that is claimed as new. The doctor contends that the cap is thus made much more readily and quickly, and, moreover, as the collar and cap are united, the little soldering that is needed to complete it will not disturb its adjustment to the root. The balance of the work, the adjustment of the dowel, etc., is the same as in the construction of any backed and soldered crown.

If it is desired to complete the crown with porcelain, soft platinum is used in place of the gold, and the tabs are turned over and hammered down to strengthen the top of the cap instead of being cut off, a little pure gold being fused over them to make them more secure.

The weak point of this device is the necessity of using pure gold or soft platinum, no other available metal being sufficiently pliable. Those who have had much experience with such work have universally found that half-collar crowns made of these soft and pliable metals are not satisfactory. Under stress the collar bends. A much more rigid metal is required to give security to the fixture and conserve the strength of the root.

Dr. Sanger says, "I have applied for a patent on the half-collar crown in order that no one else may do so and attempt to mulct the profession for the privilege of using it. I am glad to give it to you for what it is worth, and only ask in return that it shall bear my name."—W. H. T.

---

Cleansing the Hands.—Dr. Chas. H. Gerrish, of Exeter, N. H., recommends old high-grade toilet soap, the older the better, and plain Indian cornmeal, for cleansing the hands before going from the laboratory to the office.—*Dental Cosmos,* May, 1903, page 401.

